# X-ray Absorption Spectroscopy as a Process Analytical Technology: Reaction Studies for the Manufacture of Sulfonate-Stabilized Calcium Carbonate Particles

**DOI:** 10.1021/acs.iecr.3c02540

**Published:** 2023-10-02

**Authors:** Thokozile A. Kathyola, Sin-Yuen Chang, Elizabeth A. Willneff, Colin J. Willis, Giannantonio Cibin, Paul Wilson, Anna B. Kroner, Elizabeth J. Shotton, Peter J. Dowding, Sven L.M. Schroeder

**Affiliations:** † School of Chemical and Process Engineering, University of Leeds, Leeds LS2 9JT, U.K.; ‡ Diamond Light Source, Harwell Science & Innovation Campus, Didcot, Oxfordshire OX11 0DE, U.K.; § School of Design, University of Leeds, Leeds LS2 9JT, U.K.; ∥ Infineum UK Ltd., Milton Hill Business & Technology Centre, Abingdon, Oxfordshire OX13 6BB, U.K.

## Abstract

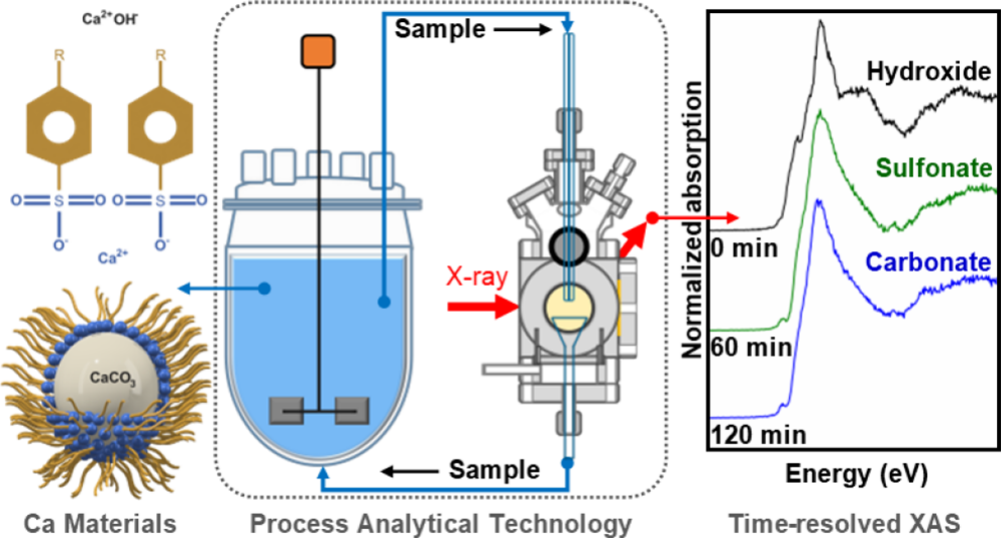

Process analytical technologies are widely used to inform process control by identifying relationships between reagents and products. Here, we present a novel process analytical technology system for *operando* XAS on multiphase multicomponent synthesis processes based on the combination of a conventional lab-scale agitated reactor with a liquid-jet cell. The preparation of sulfonate-stabilized CaCO_3_ particles from polyphasic Ca(OH)_2_ dispersions was monitored in real time by Ca K-edge XAS to identify changes in Ca speciation in the bulk solution/dispersion as a function of time and process conditions. Linear combination fitting of the spectra quantitatively resolved composition changes from the initial conversion of Ca(OH)_2_ to the Ca(R–SO_3_)_2_ surfactant to the ultimate formation of *n*CaCO_3_·*m*Ca(R− SO_3_)_2_ particles. The system provides a novel tool with strong chemical specificity for probing multiphase synthesis processes at a molecular level, providing an avenue to establishing the relationships between critical quality attributes of a process and the quality and performance of the product.

## 1 Introduction

Process analytical chemistry (PAC) tools monitor changes in the physicochemical properties during industrial synthetic processes *operando* and are part of any process analytical technology (PAT) strategy.^1,2^ PAT tools provide crucial information about the relationship between reagents and in-process materials with the desired product.^1,2^ Specifically, PAT identifies, measures, and monitors critical quality attributes (CQA) of a process in relation to the quality and performance of the final product. Analytical techniques such as infrared (IR), Raman, and ultraviolet–visible (UV–vis) spectroscopies as well as X-ray diffraction (XRD) are the main online PAT analysis across the fine chemicals and oil industries.^2−4^ Here, we describe how X-ray absorption spectroscopy (XAS) can fill gaps in the information accessible by these techniques, making it a potential PAT tool for applied R&D.

XAS is very sensitive to changes in process chemistries because it probes the local molecular structure around X-ray-absorbing atoms and can thus be applied to both ordered and disordered materials, including gas, liquid, and solid systems. Unlike XRD, its structure sensitivity is not limited by noncrystallinity. It is also much less impacted by the presence of solvents or other media that may mask the desired information as is the case with IR and Raman spectroscopy.^2,5^ It provides both qualitative and quantitative element specific information about the physical and chemical attributes of a material. For example, in the case of crystallizing systems, information that can be obtained includes kinetic and mechanistic information on nucleation, crystallization, or dissolution, crystal structure and polymorphic form, particle size, solid composition, and solute concentration.^2,4,6^ However, XAS requires an X-ray source with a tunable photon energy, which is currently available almost exclusively at synchrotron radiation facilities. This has limited the use of XAS on industrial systems and hence its consideration as a PAT tool. The past decade has seen significant steps toward the expansion of XAS provision at national synchrotron facilities all over the world, accessible both for fundamental and applied research, so that XAS is now in many fields, e.g., catalysis, considered part of the standard analytical toolkit.^7−11^ Moreover, XAS systems using laboratory X-ray sources are now readily available at a price point comparable to XRD, thus opening a realistic perspective to a wider use of XAS in applied manufacturing research.^12−15^ Wide application of these laboratory-based instruments to complex industrially relevant systems is currently limited by low energy resolution, low signal-to-noise ratio, and extended data acquisition times. However, recent studies show promising *in situ* and *operando* results that highlight the complementary aspects of laboratory and synchrotron XAS.^16,17^

Here, we demonstrate XAS monitoring of chemical transformations in a multicomponent and multiphase industrial process by use of a continuous-flow liquid-jet loop that permits windowless real-time analysis of liquid reaction mixtures that contain elements with absorption edges in the tender X-ray range, i.e., at photon energies between 2 and 7 keV. XAS monitoring of liquid systems in this photon energy range has traditionally been limited by the fact that the X-ray penetration through ambient air and through window materials on reactors and flow tubes becomes poor. We have recently shown how windowless liquid-jet monitoring of suspensions and viscous fluids with a fast flow loop constructed from widely available standard components overcomes these limitations elegantly.^18^ We have now used this setup to monitor process chemistry, investigating a scaled-down version of a complex industrial manufacturing process for the synthesis of sulfonate-stabilized calcium carbonate particles (*n*CaCO_3_·*m*Ca(R−SO_3_)_2_) .^19−21^ Our study highlights the sensitivity of XAS to the structure of the calcium-containing materials in dilute dispersions, which becomes evident through the *operando* X-ray absorption near-edge structure (XANES) in the spectra. The final products are lubricant oil additives for deposit control and protection against corrosion in combustion engines.^22−24^ The overbasing process is a complex four-phase system involving interfacial reactions between suspended solid and dissolved calcium hydroxide (Ca(OH)_2_) in a polar methanol/water phase, akylbenzene sulfonic acid(R−SO_3_H) solution in a nonpolar organic solvent, and carbon dioxide (CO_2_) gas.^25−28^ The properties of the products formed are strongly dependent on temperature, mixing dynamics, and the composition of the gas phase. Both water-in-oil and oil-in-water emulsions can be formed, and their balance can be affected by evaporation of volatile solvents.^29^ Ultimately, particulate calcium carbonate (CaCO_3_) products are generated, which can arise in at least six distinguishable crystalline or noncrystalline (“amorphous”) forms.^30^ The final particle products contain colloidal nanometer-sized CaCO_3_ (2 to 7 nm) that is stabilized by a calcium sulfonate surfactant (Ca(R−SO_3_)_2_. The CQAs thus include the crystal structure, polymorphic form, and solid composition.

## 2 Experimental Methods

### 2.1 Materials

Ca(OH)_2_; L’hoist, U.K., Ca(R–SO_3_)_2_; Infineum, U.K., sulfonate-stabilized CaCO_3_ particles (*n*CaCO_3_·*m*Ca(R–SO_3_)_2;_ Infineum, U.K.), and four CaCO_3_ polymorphs—calcite (Sigma-Aldrich), aragonite (Alfa Aesar), vaterite, and amorphous calcium carbonate (ACC)—were used as references for the *operando* study of the overbasing process. The vaterite and ACC were synthesized using methods by Shivkumara et al.^31^ and Koga et al.,^32^ respectively. In this study, sulfonate-stabilized CaCO_3_ particles were synthesized using Ca(OH)_2_, R–SO_3_H (Infineum, U.K.), mineral oil (Infineum, U.K.), deionized water, methanol (Fisher Scientific), toluene (Fisher Scientific), and CO_2_ (Air Products). Helium (He) and nitrogen (N_2_) gases from Air Products were also used.

### 2.2 Particle Synthesis

**Figure 1:**
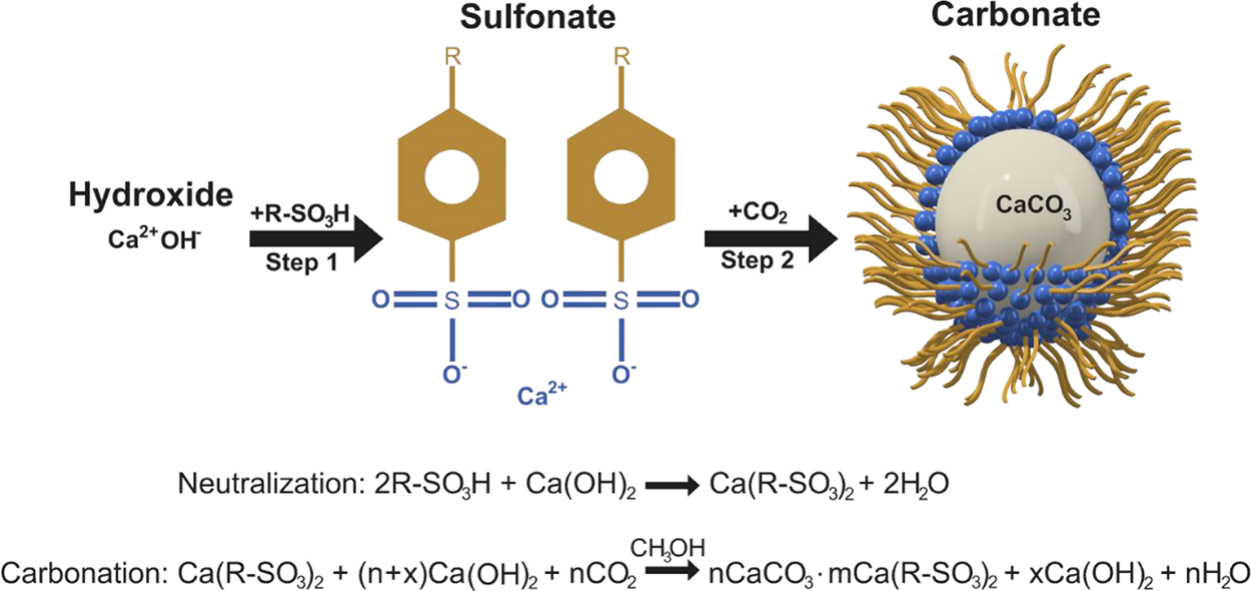
Schematic of the formation of sulfonate-stabilized CaCO_3_ particles via neutralization (step 1) and carbonation (step 2) reactions.

The synthesis of *n*CaCO_3_·*m*Ca(R–SO_3_)_2_ particles (Figure 1) was studied *operando* using a modified overbasing process similar to the methods used by Markovic et al.^19^ and Alcock.^20^ The synthesis consisted of three main stages:

Step 1: Neutralization. Reaction of R–SO_3_H acid with Ca(OH)_2_ to produce the Ca(R–SO_3_)_2_ surfactant (30 min).
Step 2: Carbonation. Reaction of Ca(OH)_2_ with CO_2_ in the presence of Ca(R–SO_3_)_2_ to form *n*CaCO_3_·*m*Ca(R–SO_3_)_2_ particles (60 min).
Step 3: Heat soak. Heating of the postcarbonation mixture to allow for reaction completion and growth of the carbonate particles and to reduce the formation of sludge and sedimentation (60 min).

Initially, methanol (280.0 g), water (27.0 g), toluene (517.0 g), and mineral oil (21.8 g) were loaded into a 1 L baffled glass reactor and stirred (at 400 rpm). Ca(OH)_2_ (4.2 g) was then added to the vessel. Neutralization (step 1) was initiated by the addition of the R–SO_3_H (170 g) and toluene (100 g) in semibatch mode. The subsequent stage, carbonation (step 2), involved the second addition of Ca(OH)_2_ (4.2 g). Approximately 95 wt % of the required stoichiometric amount of CO_2_ was then flowed through the system for an hour at 57 mL/min at 28 ±2 °C. After carbonation, during heat soak (step 3), the temperature in the reactor was raised to 60 ±2 °C max to improve product yield and filtration properties.

### 2.3 Reactor System and Process Control

**Figure 2:**
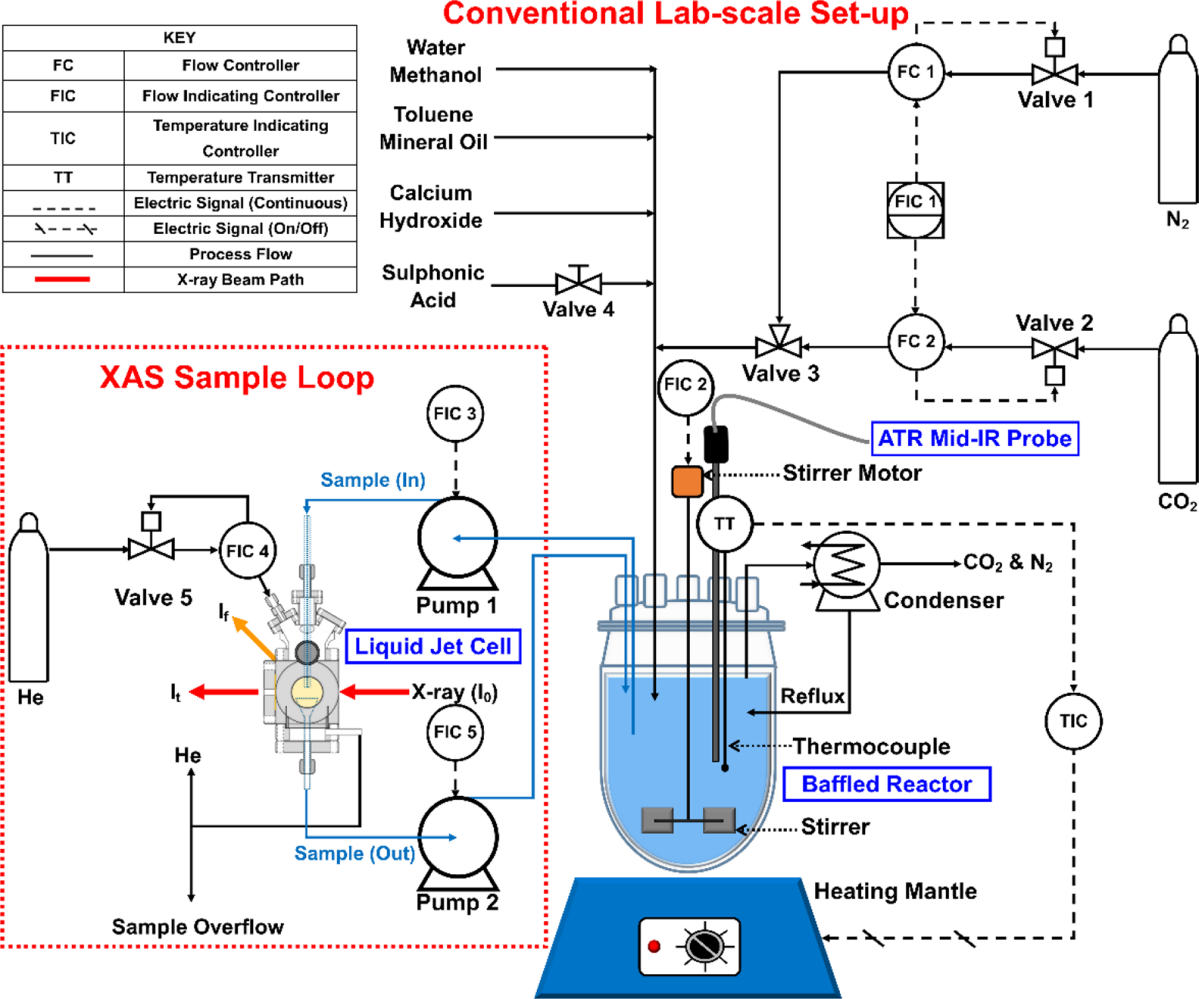
Piping and instrumentation diagram (P&ID) of the continuous-flow liquid-jet PAT experimental setup for the *operando* XAS measurements.

The particle synthesis was carried out in a 1 L glass baffled reactor using a conventional lab-scale setup (Figure 2) under a constant N_2_ (30 mL/min) environment. The sample fluid flow to and from the reactor (Figure 2 FIC 3 and 5) was controlled by two peristaltic pumps (Watson Marlow 520Du and 520S). High fluid flow rates (∼320 mL/min) through the liquid-jet sample loop were required to circumvent phase separation.

Fluctuations in the temperature of the reaction mixture must be minimized, especially during the neutralization (step 1) and carbonation (step 2) stages of the overbasing process. The exothermic reactions of R−SO_3_H acid and CO_2_ with Ca(OH)_2_ could lead to a runaway reaction. Temperature variations were minimized through control measures, which included slow carbonation over an extended period and the dropwise addition of R−SO_3_H acid (by manually opening valve 4 in Figure 2). The reactor was heated by a 1 L Cole Palmer electrothermal heating mantle. Temperature was controlled through a feedback loop consisting of a thermocouple (Figure 2 TT) and a digital West N4400 temperature controller (Figure 2 TIC).

The contents of the reactor were mixed at a high speed (400 rpm), by a Heidolph Hei-Torque Precision 100 digital overhead stirrer (Figure 2 FIC 2), to ensure even heat distribution in the reactor. This was aided by customized pseudobaffles in the form of four equally spaced depressions in the glass reactor that emulate fin-type baffles. This customized baffle configuration was chosen over conventional wall-mounted baffles to maintain the mechanical integrity of the baffles as fluid viscosity increased. The balanced axial and radial flow created by these pseudobaffles and the Rushton turbine also promoted the mixing of the gas–liquid–solid phases and thus the formation of the in-process (Ca(R–SO_3_)_2_ surfactant and CaCO_3_) and final (*n*CaCO_3_·*m*Ca(R–SO_3_)_2_ particle) products.

All gas flows were regulated with flow controllers labeled FC 1, FC 2, and FIC 4 in Figure 2. The reactor vessel was continuously flushed with gaseous N_2_ to prevent the reaction between Ca(OH)_2_ and atmospheric CO_2_. A glass spiral reflux condenser prevented evaporation of volatile solvents and vented CO_2_ and N_2_ gas, which had been bubbled through the reactor. The XAS cell was flushed with He because it has a low X-ray absorption cross section.

### 2.4 XAS Data Collection

Ca K-edge (4038.5 eV) XAS experiments were carried out at B18, the quick EXAFS beamline at the Diamond Light Source, using a previously described liquid-jet loop.^18,33^ A Si (111) double-crystal monochromator was used. The 3 GeV synchrotron facility operated at a current of 300 mA. The beam size was 800 μm in height and 600 μm width. XANES spectra were collected every minute during each stage of the overbasing process for a duration of ∼4.5 h. Ten scans were taken before (pre-) and after (post-) each synthesis stage. These *operando* XAS data were collected in fluorescence yield (FY) using a 4-element SII Vortex silicon-drift detector with XSPRESS3 electronics. For each reference sample, a total of 10 spectra were acquired in He-flow total electron yield (TEY) mode. All experimental data were processed and analyzed using Athena in the Demeter software package.^34^ Linear combination fitting (LCF) was performed on the *operando* spectra over a range of 4020 to 4090 eV. The *ex situ* spectra of the Ca(OH)_2_, four CaCO_3_ polymorphs, Ca(R–SO_3_)_2_ surfactant, and *n*CaCO_3_·*m*Ca(R−SO_3_)_2_ particle reference samples were used as standards for the LCF.

## 3 Results and Discussion

### 3.1 PAT Sampling Loop

**Figure 3:**
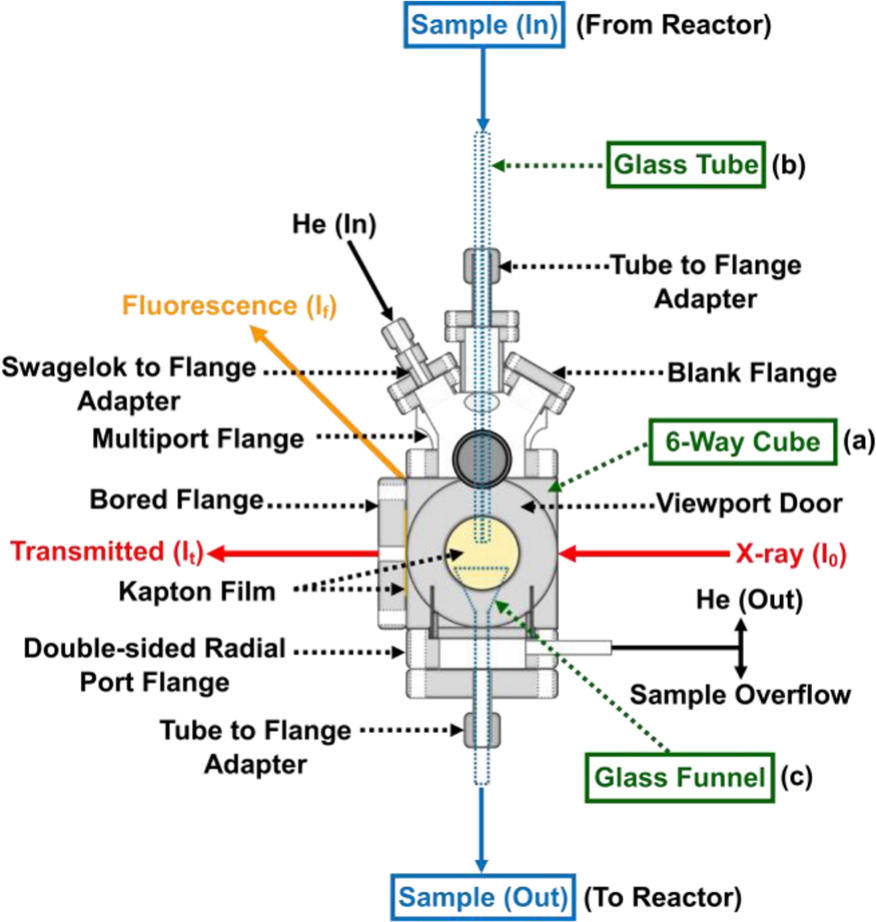
Detailed schematic of the liquid-jet cell used in the XAS PAT setup.^18^ Main cell components: (a) stainless steel 6-way Conflat flange cube, (b) glass tube, and (c) funnel (green). The entry and exit of the sample fluid (blue) and X-ray beam (red) are also highlighted.

The continuous-flow liquid-jet PAT system (Figure 2) integrates the previously described liquid-jet cell in a scaled-down industrial process in a 1 L baffled reactor (Figure 3).^18^ The baffled reactor contained a polyphasic dispersion in which the overbasing synthesis process took place (details in Section 2.1). To monitor the chemical state changes of calcium during the overbasing process with XAS in real time with minimal disruption to the synthesis, a sampling loop via the liquid-jet cell was introduced into the baffled reactor (Figure 2). Aliquots of the polyphasic dispersion were continuously pumped from the baffled reactor into the liquid-jet cell at a flow rate of ∼320 mL/min using two peristaltic pumps for the entire duration of the experiment (∼4.5 h). The inclusion of the sample loop made it possible to use the apparatus typically used for the lab-scale industrial overbasing process with only minor modifications to the synthesis process and eliminated the need to design a reactor system specifically for XAS measurements. This is particularly important because product composition is highly dependent on the experimental setup. If a significantly modified synthesis method and setup had been used, the XAS results would have been unrepresentative of the industrial process.

The liquid-jet cell consists of three main components (Figure 3).^18^ (i) A stainless steel ConFlat 6-way cube sample chamber (40 × 40 × 40 mm^3^) with a controlled He environment accommodates sample flow in the vertical direction from top to bottom, transmission of the X-ray beam in the horizontal direction from right (I_0_) to left (I_t_), and fluorescence-yield (I_f_) XAS via a detector placed perpendicular to the path of the X-ray beam; (ii) A glass tube (inner diameter = 0.8 mm) creates the liquid jet that is collected by (iii) a glass funnel (diameter = 25 mm) for recycling back to the reactor.

The liquid-jet cell was connected to the reaction vessel via Viton tubing attached to the ends of the glass tube and funnel. Viton tubing was chosen to connect the baffled reactor to the sample loop because it is sufficiently robust to tolerate the pressures induced by the high fluid flow and exposure to organic solvents over an extended period. The flow rate of sample through the liquid-jet sampling loop was regulated by digital flow controllers (FIC 3 and 5 in Figure 2) attached to the two peristaltic pumps. For the systems studied in this paper, a flow rate greater than 280 mL/min was required to create a steady liquid-jet stream ideal for XAS measurements. The high flow rate also ensured high turnover of the sample at the X-ray beam, which minimized the potential for beam damage or X-ray radiation-induced reactions.

### 3.2 *Operando* XAS Study of the Overbasing Process

**Figure 4:**
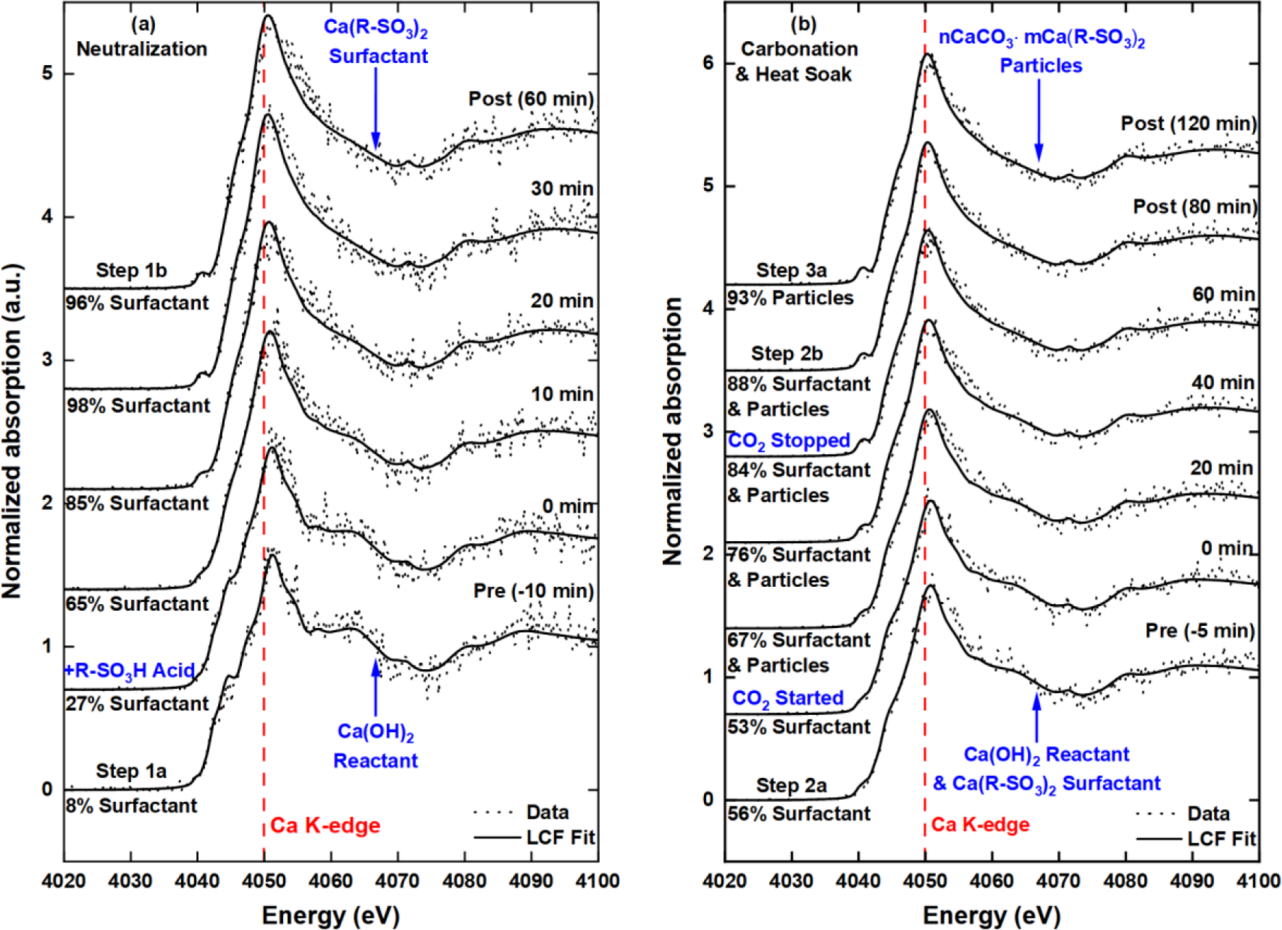
*Operando* Ca K-edge FY XANES spectra of the overbasing process. Time-dependent spectra during the (a) neutralization stage (step 1): reaction of Ca(OH)_2_ (base) and R–SO_3_H (acid) to form Ca(R–SO_3_)_2_ (surfactant) and (b) the carbonation stage (step 2): reaction of a Ca(OH)_2_ and Ca(R–SO_3_)_2_ mixture with CO_2_ to form *n*CaCO_3_·*m*Ca(R–SO_3_)_2_.

**Figure 5:**
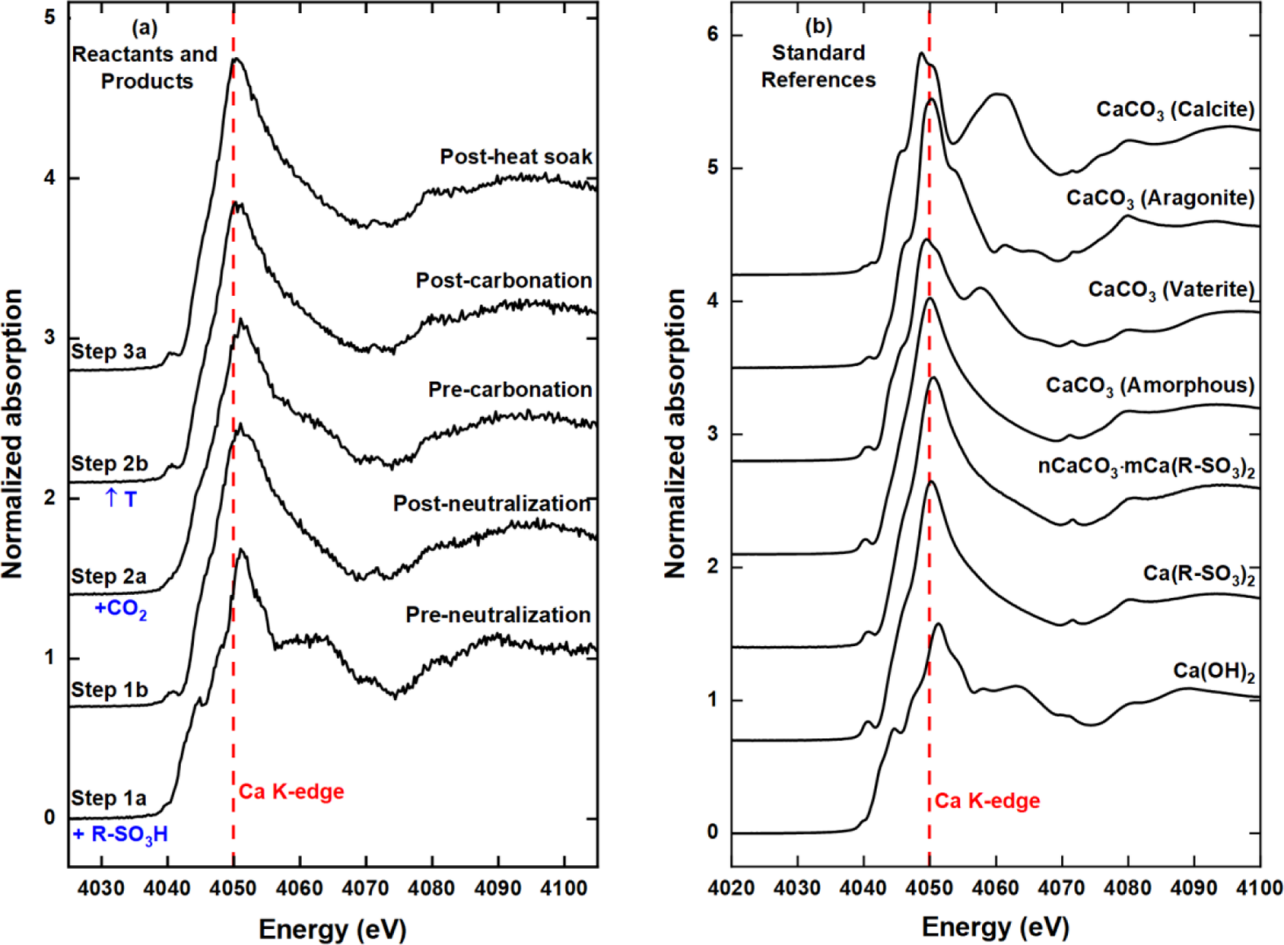
(a) Averaged *operando* Ca K-edge FY XANES spectra (of 10 scans) collected at equilibrium points between individual steps of the overbasing process and (b) *ex situ* TEY reference spectra of the Ca(OH)_2_ (reactant), Ca(R–SO_3_)_2_ (surfactant), *n*CaCO_3_·*m*Ca(R–SO_3_)_2_, and four CaCO_3_ polymorphs.

The capability of the continuous-flow liquid-jet setup (Figure 2) as a PAT tool was explored with an *operando* XAS study of the synthesis of sulfonate-stabilized CaCO_3_ particles. XAS was employed to determine Ca(OH)_2_ dissolution, CaCO_3_ crystallization, and polymorphic form, the effects of the surfactant on the electronic structure of the particles, and composition of the reactants and products over time. FY XANES spectra were collected at the Ca K-edge during the overbasing process (Figure 4) and while dispersions were at equilibrium between individual steps of the overbasing process (Figure 5a). Results from each of the three main steps of the particle synthesis, i.e., neutralization (step 1), carbonation (step 2), and heat soak (step 3), are discussed separately below.

#### 3.2.1 Neutralization (Step 1)

The initial dispersion contains solid Ca(OH)_2_ in water, methanol, toluene, and mineral oil. The Ca(R–SO_3_)_2_surfactant was prepared by the slow addition of R–SO_3_H acid to the Ca(OH)_2_ dispersion over a period of 30 min. The XANES spectrum of the preneutralization dispersion (step 1a, Figure 4a and 5a) is identical to the *ex situ* reference spectrum of the solid phase Ca(OH)_2_ reactant (Figure 5b). This confirms that the calcium species in the dispersion are solely present as Ca(OH)_2_. Following the complete addition of R–SO_3_H, the postneutralization (step 1b) spectrum indicates the presence of Ca(R–SO_3_)_2_ “in-process” product. The transformation from Ca(OH)_2_ (step 1a) to Ca(R–SO_3_)_2_ (step 1b) is readily evident from changes in the XANES spectra from 4040 to 4060 eV (Figures 4a and 5a). The absorption bands in the spectra are due to single-/double-electron dipole transitions from 1s/1s3p core states to unoccupied 3d and 4p states in the valence region. The 1s → 3d transitions arise at ∼4040 eV (pre-edge) followed by 1s → 4p electronic transitions from 4045 to 4060 eV. The intensity of the peak at ∼4050 eV can be correlated to the scattering of the photoelectron emitted from the central Ca absorber off of oxygen neighbors in the first Ca–O shell.^35−37^ Whereas the postedge peak at ∼4058 eV is influenced by interactions between Ca 4p states in the absorbing Ca atom and Ca 3d and 4s states in neighboring Ca atoms.^38,39^ In Figure 4a, the features at 4045 and 4060 eV are particularly diagnostic of changes in the local environment around Ca. Changes in these features can be attributed to the dissociation of the Ca^2+^ and OH^–^ ions in water and the subsequent formation of ionic bonds between the Ca^2+^ and R–SO_3_^–^ ions.

**Figure 6:**
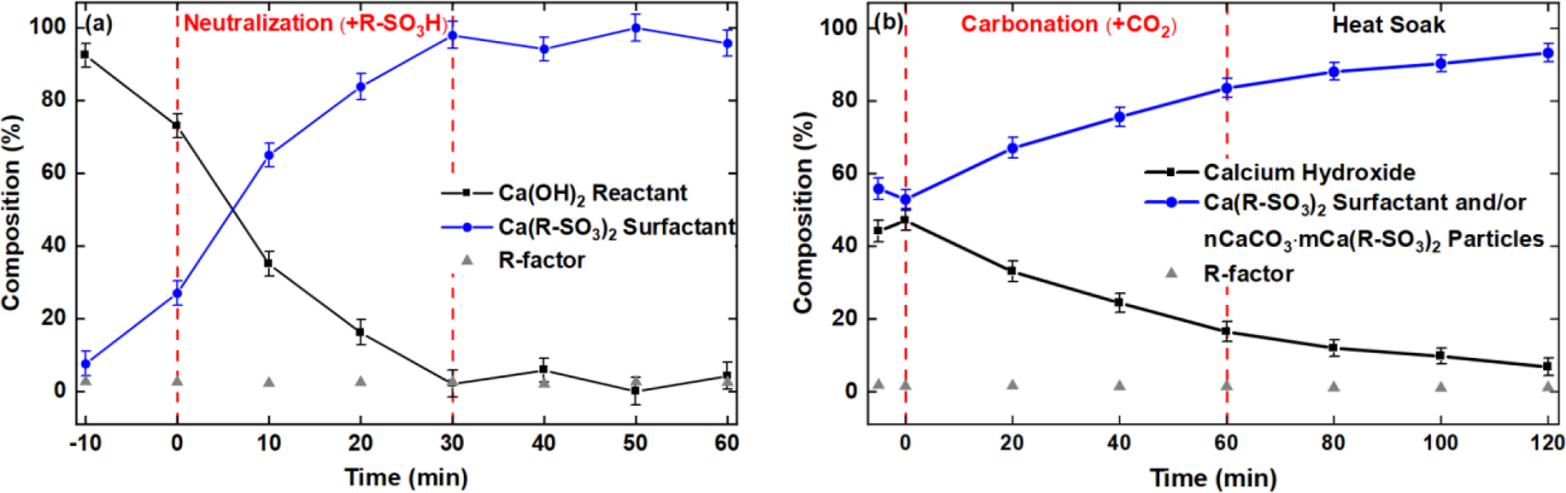
LCF results of the calcium-centric materials (Ca(OH)_2_ reactant, Ca(R–SO_3_)_2_ surfactant, and *n*CaCO_3_·*m*Ca(R–SO_3_)_2_ present in the polyphasic dispersion during the (a) neutralization (step 1) and (b) carbonation (step 2) and heat soak (step 3) reactions of the overbasing process.

Linear combination fitting (LCF) was performed on the *operando* XANES spectra of the dispersion using the reference spectra from solid Ca(OH)_2_ and Ca(R–SO_3_)_2_, as shown in Figure 5. LCF shows that the Ca(R–SO_3_)_2_ present in the dispersion increased from about 8(3) to 96(4)% as the Ca(OH)_2_ content decreased (Figure 6a). LCF slightly underestimated the intensity of features present in the experimental spectra between 4050 and 4060 eV after 10 min of reaction (Figure 4a), highlighting the presence of Ca with a different local environment to that of the Ca(OH)_2_ and Ca(R–SO_3_)_2_ references. The third component that has not been taken into account in the LCF is likely to be hydrated Ca^2+^. This hypothesis is primarily based on the fact that water is formed as a byproduct during the neutralization stage (Section 2.1). The broad 1s → 4p feature at 4050 eV in the postneutralization spectrum edge feature is similar to that of a 6 M CaCl_2_ solution containing fully hydrated Ca^2+^.^37^ Future quantitative LCF analysis of the neutralization step will require reference spectra of an aqueous solution of Ca(OH)_2_ and/or CaCl_2_.

#### 3.2.2 Carbonation (Step 2)

After neutralization (step 1), a dispersion of Ca(R–SO_3_)_2_ surfactant (Figure 4a and 5a, postneutralization) is present in the baffled reactor. A second charge of solid Ca(OH)_2_ is added to this dispersion immediately prior to carbonation. The presence of Ca(OH)_2_ in the dispersion is readily evident from the Ca K-edge XAS (precarbonation, Figure 4b and 5a). The dispersion spectrum changes from being smooth and relatively featureless (postneutralization) to a spectrum with some defined 1s → 4p features at 4060 eV (precarbonation). This confirms that the dispersion contains a mixture of unreacted solid Ca(OH)_2_ and neutral Ca(R–SO_3_)_2_ surfactant.

Quantitative LCF analysis (Figure 6b) of the precarbonation spectrum in Figure 4b shows that the dispersion consists of 44(3) and 56(3)% of Ca(OH)_2_ and Ca(R–SO_3_)_2_ surfactant, respectively. Following the introduction of CO_2_ flow, the composition of the hydroxide decreased with time as the *n*CaCO_3_·*m*Ca(R–SO_3_)_2_ particles formed (Figure 6b). The LCF shows that after the 60 min carbonation reaction, more than 80% of the calcium-centric material present in the postcarbonation dispersion (Figure 4b) is the *n*CaCO_3_·*m*Ca(R–SO_3_)_2_ particles. Due to the similarity of the Ca(R–SO_3_)_2_ surfactant and the *n*CaCO_3_·*m*Ca(R–SO_3_)_2_ particles XANES spectra (Figure 5b), the percentages calculated by LCF using the particle reference spectrum have been attributed to both components. Future measurements using C K-edge XAS could help distinguish the particles and surfactant based on visible contributions from carbon present in CO_3_^2–^, which would only be present in the particles spectra.^40^

#### 3.2.3 Heat Soak (Step 3)

The spectra of the final steps in Figure 4b—postcarbonation (step 2b) and postheat-soak (step 3a)—show the presence of the final product, i.e., the *n*CaCO_3_·*m*Ca(R–SO_3_)_2_ particles. During heat soak, process conditions are the same except that the reactor temperature is increased from 28 to 60 °C. There were no significant changes in the spectra (Figures 4b and 5a) of the dispersion during the 90 min heat soak step. However, the LCF analysis (Figure 6b) of the time-resolved XANES spectra showed that the remaining CO_2_ dissolved in the system continued to react with Ca(OH)_2_ after the CO_2_ flow was terminated. This was deduced from a slight increase in the particles/surfactant content from 88(2)% (Figure 4b, postcarbonation) to the expected 93(2)% within 20 min of the heat soak stage. 100% conversion to particles/surfactant is not expected as the amount Ca(OH)_2_ that was added before carbonation (Figure 4b, precarbonation) was 5% in excess compared to the required stoichiometric amount.

As one progresses sequentially through the individual reaction steps, the number and intensity of the 1s → 4p pre- and postedge features in the XANES (Figures 4a and 5a) decrease such that the spectrum of the *n*CaCO_3_·*m*Ca(R–SO_3_)_2_ product is relatively featureless compared to the spectrum of the Ca(OH)_2_ starting material. This lack of features has previously been associated with local disorder around the calcium atom^41,42^ and is reminiscent of the XANES spectrum of amorphous calcium carbonate (ACC) (Figure 5b). The centrosymmetric polymorph of CaCO_3_ calcite has the most prominent 1s → 4p features (Figure 5b), suggesting that the particle CaCO_3_ core is not calcite. There is a possibility of the core being aragonite or vaterite (based on the presence of a 1s → 3d transition feature at 4040 eV), whereby the 1s → 4p features related to crystalline CaCO_3_ are being masked. This hypothesis is supported by the similarity of the Ca(R–SO_3_)_2_ surfactant (postneutralization) and *n*CaCO_3_·*m*Ca(R–SO_3_)_2_ particles (postcarbonation) XANES (Figure 5). It can be postulated that the (surface) Ca of the surfactant outer layer of the particle reverse micelles makes up a significant fraction of the total signal such that it masks the signal of the very small (1 to 5 nm) CaCO_3_ particles at the particle center. Previous XAS studies^21,23,40−43^ on similar particles have not compared the particle and surfactant, and further work is required to resolve this point. Notably, the presence of CaCO_3_ in the particle product is supported by a shift of the absorption edge by ∼−1 eV from preneutralization (step 1a) (*t* = 0 min) to postheat soak (step 3a) (*t* ∼ 180 min) as a function of reaction time (Figures 4a and 5a). This shift mirrors trends observed in reference spectra where, as one moves from Ca(OH)_2_ through the polymorphs of CaCO_3,_ a maximum shift of ∼ −2.5 eV is observed for calcite (Figure 5b).

## 4 Conclusions

The applicability of XAS as a PAT tool was demonstrated by a Ca K-edge *operando* study of surfactant-stabilized CaCO_3_ particle synthesis. A novel continuous-flow liquid-jet PAT setup was applied to study the multiphase overbasing process containing two liquid phases, a gas, and a solid phase with minimal modifications to a conventional lab-scale reactor. The results demonstrate the ability of XAS to sensitively capture information on process chemistry on the nanoscale. Real-time changes were evident in the Ca K-edge XAS spectra of the polyphasic dispersion during the neutralization and carbonation stages of the synthesis. In both cases, qualitative and quantitative analysis of the XANES regions showed the conversion of Ca(OH)_2_ to the Ca(R–SO_3_)_2_ surfactant and *n*CaCO_3_·*m*Ca(R–SO_3_)_2_ particle products as a function of time. XAS was capable of simultaneously differentiating up to three different local environments present in the dispersion. It also identified the probable presence of hydrated Ca^2+^ in a dispersion containing the Ca(OH)_2_ and Ca(R–SO_3_)_2_ surfactant. Finally, the quantitative analysis of the XANES spectra with LCF demonstrated how dissolution rates of materials with low solubility such as Ca(OH)_2_ can be determined. Determination of *operando* reaction kinetics using XAS would also be beneficial to other industrial sectors including agrochemicals, pharmaceuticals, and energy materials. Notably, the liquid-jet PAT system has recently been used in a soon-to-be published study on hybrid hydrogen–manganese redox flow batteries and regenerative fuel cells at the titanium and manganese K-edges.^44^ Further development is required to allow for analysis in the lower tender X-ray region (<4 keV), which includes absorption edges such as phosphorus and sulfur.
